# Randomised controlled trials of complex interventions

**DOI:** 10.1186/1745-6215-12-S1-A142

**Published:** 2011-12-13

**Authors:** John Norrie

**Affiliations:** 1 Centre for Healthcare Randomised Trials, Health Services Research Unit, Health Sciences Building, University of Aberdeen, UK

## 

Although all randomised controlled trials (RCT) share much in common in terms of design, conduct, analysis and reporting, trials of complex interventions (loosely “interventions with several interacting components”) can have special issues which need appropriate consideration to deliver high quality and credible evidence to change or inform practice.

For example, blinding the intervention may be impossible (a surgical compared with a medical intervention), and standardising the intervention (as would be obligatory and easy in a drug trial) difficult to define and harder still to achieve (for example, reaching a therapeutic alliance between patient and therapist in a cognitive behavioural therapy RCT). In addition, often complex intervention trials have patient reported primary outcomes (for example, quality of life) that present challenges (e.g. deaths, missing data). Safety reporting in complex intervention trials often presents particular challenges, struggling to adapt methodologies designed and developed for regulatory drug trials.

Designers of complex intervention trials may be forced to make assumptions about critical features of the intervention (e.g. intensity, and duration) and how to measure the treatment effect without the funds or the time to develop these aspects in a similar manner to the Phase I-IV development of drug trials. Frequently complex intervention trials are underpowered to detect important effects due to their expense and perceived difficulties. Often times one is left wondering whether the failure of a complex intervention trial to detect a treatment effect was down to sub optimal design, rather than strong & robust evidence that it does not work.

This talk will highlight various methodological challenges in the various stages of the design and running of complex intervention trials, including the development of evidence based interventions and outcomes, through to interpretation of often difficult analyses and convincing skeptical peers and journals of the robustness of findings. The talk will be illustrated by practical examples from many complex intervention trials the speaker has been involved in, including several large, multicentre, pragmatic trials funded by the NIHR/HTA, and more explanatory trials funded by MRC/EME. It will also draw on first-hand experience of sitting as an independent statistician / methodologist / trialist on various Trial Steering Committees and Data Monitoring Boards, as well as on various funding panels and journals.

